# Essential Oil Extracted from *Cymbopogon citronella* Leaves by Supercritical Carbon Dioxide: Antioxidant and Antimicrobial Activities

**DOI:** 10.1155/2019/8192439

**Published:** 2019-01-02

**Authors:** Hong Wu, Jilie Li, Yuan Jia, Zhihong Xiao, Peiwang Li, Yixian Xie, Aihua Zhang, Rukuan Liu, Zewen Ren, Mengrui Zhao, Chaozhen Zeng, Changzhu Li

**Affiliations:** ^1^Key Laboratory of Cultivation and Protection Co-constructed for Non-wood Forest Trees, Ministry of Education, Central South University of Forestry and Technology, Changsha 410004, China; ^2^Key Laboratory of State Forestry Administration on Utilization Science for Southern Woody Oil Resource, Hunan Academy of Forestry, Changsha 410004, China; ^3^Hunan Engineering and Technology Research Center of Lipids, Changsha 410004, China

## Abstract

To improve essential oil quality, especially to reserve the thermal instability of compounds, supercritical CO_2_ extraction (SFE) was applied to recover essential oil from *Cymbopogon citronella* leaves. A response surface methodology was applied to optimize the extraction process. The highest essential oil yield was predicted at extraction time 120  min, extraction pressure 25  MPa, extraction temperature 35°C, and CO_2_ flow 18  L/h for the SFE processing. Under these experimental conditions, the mean essential oil yield is 4.40%. In addition, the chemical compositions of SFE were compared with those obtained by hydrodistillation extraction (HD). There were 41 compounds obtained of SFE, while 35 compounds of HD. Alcohols and aldehydes were the main compositions in the essential oils. Furthermore, the antioxidant activities and antimicrobial of essential oils obtained by HD and the evaluated condition of SFE were compared. Results showed that the antioxidant activities of SFE oil are better than those of HD. Minimum inhibitory concentrations (MICs) were determined by the microdilution method. Essential oil obtained from SFE and HD exhibited a significant antimicrobial activity against all tested microorganisms. It is confirmed that the SFE method can be an alternative processing method to extract essential oils from *Cymbopogon citronella* leaves.

## 1. Introduction

*Cymbopogon citronella* Stapf (*C. citronella*), also known as lemongrass, belongs to *Cymbopogon* Spreng. family. *C. citronella* is a perennial grass that is widely cultivated in tropic and subtropic regions of the eastern hemisphere, such as China [1, 2]. *C. citronella* is barren and drought resistant and can tolerate a temperature range from −25°C to 40°C, suggesting that it can be planted in a wide range of environmental conditions [3]. The essential oil of *C. citronella* has been studied widely; Sriramavaratharajan et al. [3] has found that the essential oil was mainly distributed in the *C. citronella* leaves. In other research studies, the components of the *C. citronella* essential oil were identified, including mainly citral (aldehydes geranial + neral) and terpenes (myrcene-monoterpene and geranial-terpenic alcohol) [1, 2]. As an economic oil crop, the fragrance odour together with the various pharmacological activities promote the essential oil widely used as a flavor enhancer in food, perfumery, soap, cosmetic, pharmaceutical, insecticide industries, etc. [4, 5]. For example, the citral from the essential oil could be used as a raw material to prepare vitamins (A, E, and K), ionone, methylionone, and perfumes [1, 6]. Consequently, this essential oil-producing crop has attracted huge economic interests in China and abroad in recent years.

However, the *C. citronella* essential oil has always been obtained by hydrodistillation extraction (HD) method. Actually, the HD is a widely used method to measure the yield of thermally stable essential oil even today. Various researchers have extracted the essential oil from different oil crops with HD [1, 2]. Other studies [1, 2] extracted the essential oil (of 0.03% feedstock) from *Artemisia capillaris* T. by HD. Huang [7] extracted 0.25% and 1.75% of the essential oil from *Cinnamomum cassia* P. and *Zingiber officinale* R. by the HD. 2.69% of essential oil was extracted from *C. citronella* by the HD with the response surface method (RSM) reported in [1, 2]. In addition, Sargenti and Lanças [2] performed and reviewed the relative analysis for extraction efficiently of essential oil with various methods, indicating HD can be widely used as a common processing for most thermally stable essential oils. However, many research studies had reported that the high temperature and long treatment time in extraction processing contributed to degradation of thermosensitive compounds and need a great amount of energy consumption [8]. Thus, it is necessary to continue to search and establish possible alternative process to further improve essential oil quality. Furthermore, in considering the subsequent separation of the essential oil in extraction processing, our group proposed the method of supercritical fluid extraction (SFE) in this study.

As a newly and environmentally separating technology, SFE, also referred to as pressurized extraction, employs environmental-friendly agents, such as CO_2_ and water, as the mediator. SFE is an effective and selective sample separation technique, which has been widely used in industrial production, environmental analysis, and analytical chemistry [9, 10]. Several studies have demonstrated that the SFE process was applied in extracting bioactive compounds, such as flavonoids, active polysaccharide, protein, polyphenol, and essential oil [9]. Compared with various other extraction methods, Pavela [1] and SargentiLanças [2] found that the SFE was efficient in selectively fractionating the essential oil under mild extraction conditions. They also evaluated the effect of different factors on extraction yield of essential oil by SFE, resulting in 1.51% of essential oil [1, 2]. To our knowledge, nevertheless, there was no report in the literature on using SFE in extraction of essential oil from *C. citronella.*

In addition, antioxidant and antimicrobial chemistry are interesting topics at present because they play a pivotal role in pathogenesis of cardiovascular diseases, neural disorders, cancer, and aging [11]. Consequently, a majority of the reports also have focused on biological characteristics and compositions of essential oil extracted from various other plants [4, 8, 11, 12], such as *Rosmarinus officinalis*, *A. capillaris*, and *Z. officinale*, but there was few information about the essential oil of *C. citronella* leaves essential oil in the literature.

The objective of this study was therefore to optimize the SFE conditions so as to obtain the highest essential oil yield from *C. citronella* leaves. In this study, the supercritical carbon dioxide was employed to extract the essential oil, and the operational parameters were optimized using response surface methodology. The chemical compositions of the essential oil obtained from *C. citronella* leaves were also identified by gas chromatography mass spectrometry (GC-MS) analysis. Furthermore, the antioxidant activities and antimicrobial activities of the essential oil extracted by SFE were compared with those obtained by HD.

## 2. Materials and Methods

### 2.1. Materials

Fresh *C. citronella* leaves were collected from a local farm in Changsha, Hunan, soon after harvesting, and then transported and preserved in our laboratory. The leaves were air-dried (6% of moisture) and milled into powders using a frozen broken molecular machine (SPEX SamplePrep, USA). Powders were screened through an 80-mesh sieve and kept in a refrigerator at 4°C until further use.

### 2.2. Hydrodistillation (HD) Process

100 g of dried milled powder was immersed in 300 mL water and distilled for 3 h, using a Clevenger-type apparatus which was found to be sufficient for completing the extraction process. The essential oil was collected and was then weighed. The collected oil was dried with a small amount of anhydrous sodium sulfate and refrigerated prior to further analysis.

### 2.3. Supercritical Fluid Extraction (SFE) Process

The extraction processing was conducted in a 500 mL extraction vessel (Hunan Supercritical Extraction Company Ltd., Jiangsu province). The extraction was according to S. R. Sargenti [2] with minor modification. Briefly, a 200 g dried substance would be placed in an extraction kettle, including extractor and separator would be heated to 30°C and 25°C, respectively. CO_2_ is cooled in a condenser and filled into the extractor. The pressurization was conducted to maintain the pressure at 20 MPa by filling the CO_2_; thereafter, the flow of CO_2_ was adjusted to 20  L/h for essential oil by circulatory extraction. After extraction, the separation temperature and pressure were controlled to 25°C and 4 MPa, respectively. To evaluate the effect of the variables on the extraction yield of essential oil, those factors, including the extraction time, extraction temperature, and CO_2_ flow, were studied, respectively, with single-factor experimental. The standard deviation is less than 4.0%.

### 2.4. Optimization of SFE

Based on the single-factor study, the optimized experimental conditions for maximum extraction of essential oil from *C. citronella* leaves using supercritical CO_2_ were determined using the design-expert V8.0.6. Given the influence of the above variables, the extraction processing of essential oil was optimized with a Box–Behnken design (BBD) and response surface methodology (RSM) [13]. For the BBD experiments of 4 factors, there were 24 experiments augmented and 3 replications at the center values (zero level). The levels of each factor and the design table are given in Tables 1 and 2.

Design-expert V8.0.6 was used for the experiment designs and subsequent regression analysis of the response data. Statistical analysis of the model was performed to evaluate the analysis of variance (ANOVA). The quality of the polynomial model equation was judged statistically using the coefficient of determination *R*^2^ and adjustment *R*^2^, and its statistical significance was determined by the *F* value and *P* value. The significance of the regression coefficients was tested by some parameters, such as coefficient of variation (CV) and adequate precision.

### 2.5. GC-MS Analysis

The compositions of essential oil obtained by HD and SFE (in evaluated condition) were analyzed by a GC-MS analysis. The examination was performed on a Scion SQ (Bruker, USA) equipped with an 8400 autosampler. Samples were injected into the capillary column in the split and splitless modes. A 1 *μ*L aliquot of test solution was injected into a DB-5 (30 m × 250 *μ*m × 0.25 *μ*m, Agilent, USA). The oven temperature was programmed at 5°C/min from 40°C (hold 2 min) to 100°C (hold 2 min) and then 10°C/min to 250°C (hold 2 min). The injector temperature was set at 230°C in the split and splitless modes. The flow rate of the carrier gas helium was set at 1 mL/min, the transmission line temperature was set at 200°C, and the ion source temperature was set at 250°C. The electron ionization (EI) source operated at −70 eV. The MS spectra were monitored in the full scan mode from 15 to 500 *m/z* with a scan event time of 0.3 s and a scan speed of 2000 *μ*/s. The solvent delay time was set to 6.00 min. MS data analyzed by GC/MS were processed by Analyzer Pro Software (Spectralworks Ltd., Runcorn, UK) including deconvolution of mass spectra, data collection, alignment, and normalization. Metabolite peaks were deconvoluted and collected using an area threshold of 7,000, height threshold of 1, signal to noise ratio of 3, width threshold of 0.01, scan windows of 5, and smoothing of 5. The data were aligned with a retention time window of 0.3 min.

### 2.6. Assay of Antioxidant Activities

#### 2.6.1. DPPH Assay

The free-radical scavenging activity of essential oil from *C. citronella* leaves was measured by 1,1-diphenyl-2-picryl-hydrazyl (DPPH) assay according to the method of [13]. Ascorbic acid (AA) and 2,6-di-tert-butyl-4-methylphenol (BHT) were used as positive control.

#### 2.6.2. Self-Oxidation Assay

The scavenging ability of 1,2,3-phentriol to self-oxidize was investigated according to the method with a minor modification [14]. AA and BHT were used as positive control.

### 2.7. Assay of Antimicrobial Activities

#### 2.7.1. Twofold Dilution Assay

Two bacterial strains and two fungal strains were used in this study: *Staphylococcus aureus*, *E. coli*, *Aspergillus niger*, and *Aspergillus fumigatus*. All strains were gained from Key Laboratory of Academy of Life Sciences in Central South University of Forestry and Technology, Hunan, China. The bacterial cultures were grown in the liquid medium at a suitable temperature. The bacterial strains were incubated on micrococcus, nutrient, and YM media and then cultured with shaking for 12 h at 37°C or 30°C. Two plant extracts, which were, respectively, collected within SFE and HD process, were screened for antimicrobial activity using a serial twofold dilution assay [15, 16]. The minimum inhibitory concentration (MIC) of each compound was defined as the lowest concentration that inhibited microorganism growth. Bacterial growth was evaluated visually based on the degree of turbidity. Citral was used as positive control.

#### 2.7.2. Paper Disc Diffusion Assay

The antimicrobial activities of the plant extracts and fractions were determined by paper disc diffusion assay [15, 17, 18]. The diameter of each inhibitory zone was measured (in mm). Negative controls were prepared using the same solvents employed to dissolve the plant extracts. Citral was used as positive control.

### 2.8. Analytical Procedures

All samples were prepared and analyzed in triplicate. All figures were obtained by using one of Origin 8.5, Microsoft 2010, Design-Expert 8.0.6 and onimic 9.0. To verify the statistical significance of all parameters, the values of mean ± SD were calculated. The yield of essential oil obtained by HD and SFE were determined by gravimetric analysis. The extraction yield (%) of essential oil was calculated as follows:

(1) $$\begin{array}{c} Y_{1}\left(\%\right)=100\times \rho \times V_{1}, \end{array}$$

where *Y*_1_ is the extraction yield (%); *ρ* is the destiny of the essential oil; and*V*_1_ is the volume of the essential oil. The capability of scavenging the DPPH radical was calculated using the following equation:

(2) $$\begin{array}{c} I\left(\%\right)=\left[\frac{1-\left(A_{i}-A_{j}\right)}{A_{\text{c}}}\right]\times 100, \end{array}$$

where *A*_c_ is the absorbance of the bland; *A*_*i*_ is the absorbance of the sample reacted with the DPPH; and *A*_*j*_ is the absorbance of the sample. The scavenging ability of 1,2,3-phentriol of essential oil to self-oxidize was calculated using the following equation:

(3) $$\begin{array}{c} Y_{2}\left(\%\right)=\left(\frac{1-S_{1}}{S_{2}}\right)\times 100, \end{array}$$

where *Y*_2_ is the scavenging ability; *S*_1_ is the slope of the sample; and *S*_2_ is the slope of control.

## 3. Results and Discussion

### 3.1. Determining Levels for Independent Variables

**Figure 1:**
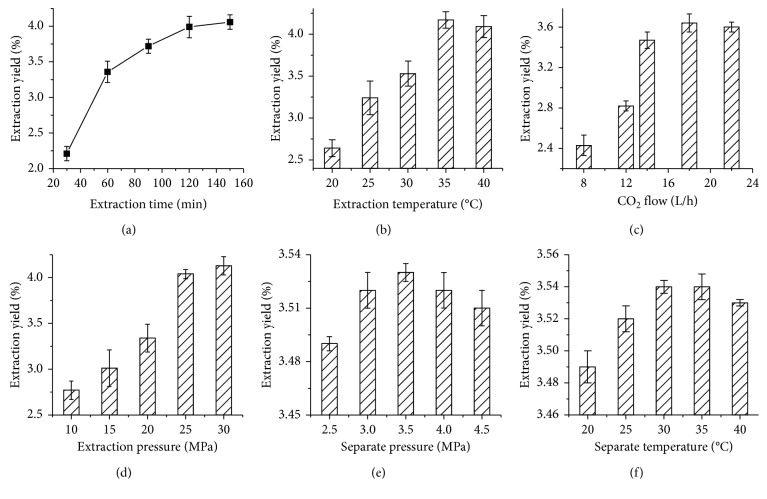
Influence of various parameters on SFE efficiency for essential oil.

In recent reports, many researchers have indicated the efficiency of extraction would be impacted significantly by some factors in SFE processing, such as extraction time, extraction temperature, and CO_2_ flow, which were respectively studied to evaluate the impact of parameters on the SFE; the results are shown in Figure 1. Firstly, the effect of different extraction time periods on the extraction essential oil yield was examined at 30, 60, 90, 120, and 150 min in the process of SFE. The essential oil produced during SFE with respect to extraction time on extraction yield is shown in Figure 1(a). Results in the extraction yield of essential oil was increased with the extraction time going up from 30 min to 150 min. When the extraction time was shorter than 60 min, the oil was extracted significantly from the *C. citronella* but the yield was still at a lower level. At 120 min, the extraction yield of essential oil reached 4.0% (w/w on origin feedstock). Thereafter, the extraction yield was almost stabilizing with the extension of time, which would increase the cost of SFE treatment. Therefore, it is favorable to choose the range of 110–130 min as a desirable holding time for the SFE of essential oil.

Extraction temperature was one of the factors affecting extraction effectiveness. According to the above results, the influence of extraction temperature at 20, 25, 30, 35, and 40°C was studied (Figure 1(b)). As seen in Figure 1(b), the extraction yield of *C. citronella* essential oil reduced significantly with the extraction temperature going up from 20°C to 40°C. Thus, there are obviously a large number of the essential oils extracted during the SFE, from 2.6% to 4.0%. The reason maybe the viscosity of supercritical fluid decreased and the diffusion coefficient of fluid increased with the elevation of extraction temperature, meanwhile the Brownian motion of essential oil in *C. citronella* was enhanced so that accelerated the processing of mass transfer, resulting in improving the dissolubility of oil [2]. At 35°C, for example, the extraction yield of essential oil increased to 4.2% (w/w in original feedstock), approximately. However, no obvious variation in extraction yield was shown after 35°C. In addition, the constituent and structure of essential oil would be degraded and destructed according to some reports [8]. Considering the economical and extraction efficiency, 32°C–38°C was chosen as a desirable holding temperature during SFE process.

The influence of CO_2_ flow of 8, 12, 14, 18, and 22 L/h is shown in Figure 1(c), which indicated that the flow of CO_2_ was a notable factor influencing the extraction results. When the CO_2_ flow increased from 8 L/h to 16 L/h, the extraction yield of essential oil increased sharply, achieving from 2.4% to 3.6%. With the increasing flow of CO_2_, nevertheless, there was an abnormal decrease in extraction yield of essential oil. Some researchers indicated that it would be conducive to the area of effective contact between feedstock and CO_2_ at the lower velocity condition so that it could be much easier to extract the essential oil from original [19]. Besides, when the flowing of CO_2_ was too higher, it would possibly lead to the inadequacy for the area of contact, but also part of extracted oil could be brought back to the extractor from the separator, in which the mixture was not separated thoroughly for the shorter separation time. It was, thus, of interest to obtain essential oil within limits of CO_2_ flow.

The effects of extraction pressure on the extraction yield of essential oil were studied with five levels at intervals of 5 MPa from 10 to 30 MPa. As illustrated in Figure 1(d), with an increase in the extraction pressure from 10 to 25 MPa, the yield of essential oil was significantly increased (from 2.7% to 4.0%). As the extraction pressure rose from 25 to 30 MPa, the extraction yield of essential oil changed little (∼4%). This was probably, according to the supercritical extraction principle reported by some researchers [2, 7, 19, 20], due to the enhancement in density and diffusivity of supercritical fluid with the increasing extraction pressure, which prompted the improvement of the strength of extraction. However, it was found that higher extraction pressure decreased the mass transfer time, even carrying part of extracted essential oil in a separator. Consequently, the extraction yield would be stabilized within higher extraction pressure. In consideration of cost, within the range of 22–28 MPa, was the appropriate extraction pressure during SFE processing in this study.

By comparison, some recent studies had reported that various factors existed in the separation reactor would have had an influence on the yield of active substance extracted from biomass during SFE processing [21]. Accordingly, some key factors, such as separation pressure and separation temperature, were evaluated for the SFE treatment in this part. As shown in Figures 1(e) and 1(f), the effect of separation pressure and separation temperature was investigated from 2.5 MPa to 4.5 MPa and 20°C to 40°C, respectively. It was abnormally apparent that the separation pressure and separation temperature resulted in an insignificant impact on extraction yield, wandering in 3.5% approximately. The result was beyond the expectation and inconsistent with previous results that the subsequent separation process would play a restrictive role on extracting essential oil, indicating the supercritical CO_2_ had an outstanding capability of efficient extraction of essential oil with SFE, which was not affected by separation pressure and separation temperature. Consequently, in consideration of cost, 3 MPa and 25°C (at room temperature) were selected to be the separation pressure and separation temperature in the next experiment.

### 3.2. Construction of the SFE Processing

After the single-factor study about the evaluation of the impact of various parameters on the SFE treatment, as shown in Figure 1, the results had revealed the various components that changed tendency in the material, including extraction time, extraction temperature, CO_2_ flow, extraction pressure, separation pressure, and separation temperature, respectively. Thereinto, the separation pressure and separation temperature were not considered in this experiment. Although a large number of essential oils had been extracted from *C. citronella* in the SFE treatment, the high efficiency needs severe treatment conditions that would increase the cost of SFE treatment. In addition, it would be also increase the cost because of interaction of various variables. Accordingly, these variables were taken into careful consideration by using Box–Behnken design (BBD) with the soft of Design-Expert V8.0.6 in this study.

The levels of each factor are given in Table 1. Twenty-seven experiments were carried out from the design (Table 2). The results of the second-order response surface model fitting for extraction yield in the form of analysis of variance (ANOVA) are given in Table 3. To test the fit of the model, the regression equation and determination coefficient *R*^2^ were evaluated. The model presented a high determination coefficient (*R*^2^ = 0.976) explaining 97.6% of the variability in the response (Table 3). The coefficients of regression were calculated and the following regression equation (4) was obtained.

Table 3 presents Fisher's *F*-test of ANOVA, which also proves that this regression model was highly statistically significant (*P* < 0.0001). At the same time, the relatively lower value of the coefficient of variation (CV = 3.25 < 10%) indicates good precision and reliability of the experiments carried out. Adequate precision for our model has a signal-to-noise ratio of 18.571 (>4) which indicates an adequate signal. The signification of each coefficient was determined by *T* and *P* which is also listed in Table 3, which demonstrates that all the linear model terms (*X*_*3*_ and *X*_*4*_) and quadratic model term (*X*_*1*_*X*_*2*_, *X*_*2*_*X*_*4*_, and *X*_*3*_*X*_*4*_) were significant. Moreover, the interactive model terms, *X*_*1*_*X*_*2*_, *X*_*2*_*X*_*4*_, and *X*_*3*_*X*_*4*_, were significant (*P* < 0.05), while the interactive model terms of *X*_*1*_*X*_*3*_, *X*_*1*_*X*_*4*_, and *X*_*2*_*X*_*3*_ were insignificant (*P* > 0.1). In addition, the empirical model (Eq. (1)) proved that the predicted data of the response from the empirical model are in agreement with the observed ones in the range of the operating variables. On the other hand, the value of adjusted (*R*^2^ = 0.948) is also very high that indicated a high significance of the model. The maximum extraction yield obtained by using above selected variables was 4.36%, and the experimental maximum obtained was 4.32 ± 0.06%. The data show that predicted data on the response from empirical model were in agreement with those observed in the range of the operating variables. The coefficients of regression were calculated, and the following regression equation was obtained:

(4) $$\begin{array}{c} Y=4.37+0.014167X_{1}+0.006667X_{2}-0.028333X_{3}-0.035833X_{4}-0.05625X_{1}^{2}+0.0425X_{1}X_{2}-0.0275X_{1}X_{3}+0.0275X_{1}X_{4}-0.0525X_{2}^{2}+0.0125X_{2}X_{3}+0.045X_{2}X_{4}-0.2975X_{3}^{2}+0.045X_{3}X_{4}-0.12875X_{4}^{2}, \end{array}$$

where *Y* = response (extraction yield), *X*_1_ = extraction time, *X*_2_ = extraction pressure, *X*_3_ = extraction temperature, and *X*_4_ = CO_2_ flow in coded values.

**Figure 2:**
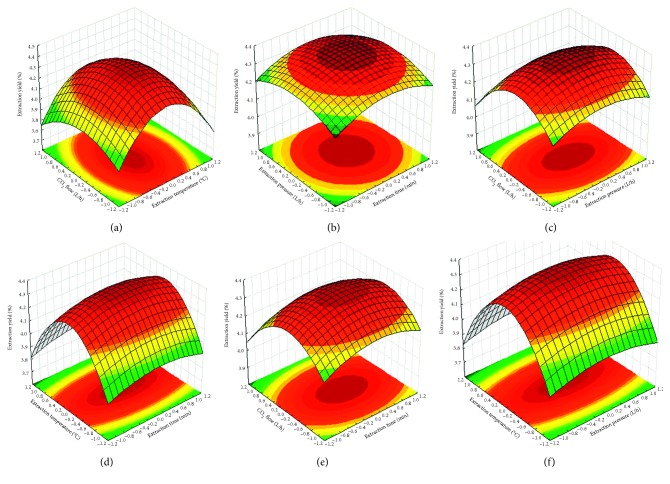
Response surface plot (3D and 2D) for the interactive effect of variables. (a) Effect of CO_2_ flow and extraction temperature with fixed extraction time and extraction pressure at 120 min and 25 Mpa, respectively; (b) effect of extraction time and extraction pressure with fixed CO_2_ flow and extraction temperature at 18 L/h and 35°C, respectively; (c) effect of CO_2_ flow and extraction pressure with fixed extraction time and extraction temperature at 120 min and 35°C, respectively; (d) effect of extraction time and extraction temperature with fixed CO_2_ flow and extraction pressure at 18 L/h and 25 MPa, respectively; (e) effect of CO_2_ flow and extraction time with fixed extraction temperature and extraction pressure at 35°C and 25 MPa, respectively; (f) effect of extraction temperature and extraction pressure with fixed CO_2_ flow and extraction time at 18 L/h and 120 min, respectively.

In order to gain a better understanding of the effects of the variables on the essential oil of extraction yield from *C. citronella* leaves, the predicted model was presented as 3D/2D response surface graphs, as shown in Figure 2, respectively, which were generated with one variable kept at its optimum level and varying the others within the experimental range (shown in Table 1). These variables were optimized as follows: extraction time 120 min, extraction pressure 25 Mpa, extraction temperature 35°C, and CO_2_ flow 18 L/h for the SFE process. Under optimized condition, the extracted essential oil reached 4.40%. Nevertheless, the result is comparable to that reported elsewhere on extraction of essential oil with various extraction methods, indicating that the SFE had significantly higher extraction efficiency and was the most energy-saving method than other methods, combining with an auxiliary instrument that could raise the cost of production [1, 5, 22–24]. Consequently, these results have suggested that the SFE treatment is effective for selectively extracting the essential oil from *C. citronella* and may be expected to use in others active components extracted from various plants in future.

Recently, many researchers argued the extraction yield and essential oil compositions, but paid little attention to the effect of extraction methods on the essential oil compositions. It would limit the development and application of essential oils [5]. Accordingly, in the next experiment, constitute features of the extracted oil are characterized with modern analytical equipment.

### 3.3. Analysis of the Extracted Essential Oil

The chemical compositions identified in the essential oils extracted by HD and SFE are summarized in Table 4. There were 35 compounds identified in HD essential oils while 41 in SFE essential oils. These compositions were grouped into seven classes (hydrocarbons, alcohols, aldehydes, esters, ketones, alkanes, and others) according to their functional groups. Alcohols and aldehydes were the main compositions in the essential oils extracted by HD and SFE. The abundant alcohols can promote the *C. citronella* leaf essential oils used for cosmetic applications. The contents of flavourless alkanes in SFE oil were much lower than those of SFE (Table 4), indicating that the essential oil in SFE contains fewer impurities, which were similar to the previous report [8]. The main component of essential oil extracted by SFE and HD was geranialdehyde and the next was geraniol, which were consistent with previous studies [23, 25]. Furthermore, the content of this major compound in SFE extracts was higher than HD. This proved that supercritical fluid did not alter the main effective components in *C. citronella* essential oil, but the content of effective composition and extraction yield of essential oil was higher [8].

### 3.4. Antioxidant Activity Analysis

In this study, antioxidant activity of essential oil from the *C. citronella* leaves was evaluated using DPPH scavenging and 1,2,3-phentriol self-oxidation activity assays. The IC_50_ values of essential oil are summarized in Table 5. As reports, 1,2,3-phentriol rapidly autoxidizes in the autoxidized solution, and several intermediate products, such as O_2_^−^, were formed, which resulted in the solution to become yellow brown with a spectrum showing a shoulder between 400 and 425 nm. The antioxidants could interfere with 1,2,3-phentriol autoxidation by acting as scavengers of O_2_^−^ [11, 12]. Therefore, antioxidant ability can be determined by the scavenging activity of self-oxidation of 1,2,3-phentriol. Table 5 shows that both essential oils, extracted by SDE and SFE, respectively, had much more effective scavenging power for self-oxidation of 1,2,3-phentriol than BHT and ascorbic acid and it should be explored as a potential antioxidant with SFE process.

Besides, the model of scavenging the stable DPPH radical is a widely used method of evaluating the free-radical scavenging ability of natural compounds. In the DPPH test, the antioxidants were able to reduce the stable DPPH radical to the yellow-colored diphenylpicrylhydrazine. The effect of antioxidants on DPPH radical scavenging was thought to be due to their hydrogen-donating ability [12]. The DPPH scavenging activity of the essential oils expressed in terms of IC_50_ is shown in Table 5, with the higher antioxidant potency of DPPH scavenging compared with the antioxidant potency of BHT and ascorbic acid. Thereinto, the essential oil extracted by SFE had the highest antioxidant potency of DPPH scavenging.

The antioxidant activities of antioxidants have been attributed to various mechanisms, and one test is normally not enough to evaluate precisely the antioxidant activity of potential antioxidant [11]. Therefore, we used these two assays to evaluate the total antioxidant capacity of essential oil from the *C. citronella* leaves. The results indicated that the essential oil had a noticeable effect on scavenging self-oxidation of 1,2,3-phentriol and were able to scavenge free radicals.

### 3.5. Antimicrobial Activity Analysis

The antimicrobial activities of extracts gained from HD and SFE had been assessed using a serial twofold dilution assay and paper disc diffusion assay, according to Hu et al. [23, 24, 26].  Table 6 shows the variation in the antimicrobial properties of the essential oil extracted from the *C. citronella* leaves. Among bacterial strains, the *E. coli* has the strongest inhibitory effect by the essential oil. Meanwhile, the essential oil shows the antifungal or anticandidal activity as well. On the other hand, more precise data concerning the antimicrobial properties of the extracts were obtained by paper disc diffusion assay [26].  Table 6 indicates the maximal inhibitory zones for each of the microorganisms that were sensitive to the extracts, which were in the range of 15–35 mm. Thereinto, the greater inhibition was observed in the case of the SFE extracts than the others. Hence, the SFE extracts was significant antimicrobial or antifungal activities against all of the microorganisms tested, except *A. niger*.

In summary, the SFE extracts have significant antimicrobial activity against *E. coli* and *A. niger*, respectively. Antimicrobial activity in essential oil depended on mostly aldehydes and acid esters [22, 27]. Recently, some literature reported there was the highest level of activity against *E. coli* with tea oil, tannic acid, and saponins [6, 20, 23]. Accordingly, these observations suggested that the antimicrobial activity of essential oil may be due to the presence of ester bond and ether bond [28]. Other phenolic acid-like phenols were also thought to contribute to plant defenses against pests and pathogens [11].

## 4. Conclusions

The essential oil was attained from *C. citronella*, a common herbaceous plant, by supercritical fluid extraction, resulting in 4.40% of essential oil extracted under mild condition. Alcohols and aldehydes were the main compositions in the essential oils extracted by HD and SFE. Compared with HD essential oils, the SFE essential oils contain fewer impurities and had better antioxidant and antimicrobial activities. Supercritical fluid extraction has presented an outstanding feature in efficient extraction of active components of the plant, which can be a desirable candidate for the current extraction processing.

## Figures and Tables

**Table 1:** Experimental range and levels of independent variables.

Variables	Symbol	Code levels		
		−1	0	1
Extraction time (min)	*X*_1_	110	120	130
Extraction pressure (MPa)	*X*_2_	22	25	28
Extraction temperature (°C)	*X*_3_	32	35	38
CO_2_ flow (L/h)	*X*_4_	16	18	20

**Table 2:** Full factorial BBD matrix of three variables in coded units and the experimentally observed response.

Runs	*X*_1_	*X*_2_	*X*_3_	*X*_4_	Extraction yield (%)
1	110	22	35	18	4.29
2	110	28	35	18	4.21
3	130	22	35	18	4.23
4	130	28	35	18	4.32
5	120	25	32	16	4.06
6	120	25	32	20	3.91
7	120	25	38	16	3.89
8	120	25	38	20	3.92
9	110	25	35	16	4.26
10	110	25	35	20	4.11
11	130	25	35	16	4.19
12	130	25	35	20	4.15
13	120	22	32	18	4.07
14	120	22	38	18	3.94
15	120	28	32	18	4.06
16	120	28	38	18	3.98
17	110	25	32	18	3.95
18	110	25	38	18	4.02
19	130	25	32	18	4.08
20	130	25	38	18	4.04
21	120	22	35	16	4.26
22	120	22	35	20	4.11
23	120	28	35	16	4.19
24	120	28	35	20	4.22
25	120	25	35	18	4.37
26	120	25	35	18	4.36
27	120	25	35	18	4.35

**Table 3:** ANOVA results for the quadratic equation for the extraction yield of essential oil.

Term	Freedom degrees	Sum of squares	Mean square	*F* value	*P*_r_ > *F*
Model	14	0.564475	0.04032	35.08174	<0.0001
*X*_1_	1	0.002408	0.002408	2.095468	0.173353
*X*_2_	1	0.000533	0.000533	0.464048	0.508671
*X*_3_	1	0.009633	0.009633	8.381873	0.013447
*X*_4_	1	0.015408	0.015408	13.40665	0.003257
*X*_1_ · *X*_1_	1	0.016875	0.016875	14.68278	0.002388
*X*_1_ · *X*_2_	1	0.007225	0.007225	6.286405	0.027545
*X*_1_ · *X*_3_	1	0.003025	0.003025	2.632024	0.130689
*X*_1_ · *X*_4_	1	0.003025	0.003025	2.632024	0.130689
*X*_2_ · *X*_2_	1	0.0147	0.0147	12.79033	0.003808
*X*_2_ · *X*_3_	1	0.000625	0.000625	0.543807	0.475032
*X*_2_ · *X*_4_	1	0.0081	0.0081	7.047734	0.020994
*X*_3_ · *X*_3_	1	0.472033	0.472033	410.7118	<0.0001
*X*_3_ · *X*_4_	1	0.0081	0.0081	7.047734	0.020994
*X*_4_ · *X*_4_	1	0.644342	0.644342	76.92326	<0.0001
Pure error	12	0.013792	0.001149	No clear	No clear
*R* ^2^ = 0.9761; Adj. *R*^2^ = 0.9483; CV = 3.25%; Adeq. precision = 18.571					

**Table 4:** Chemical compositions of essential oils.

No.	Components	Concentration of essential oil (%)	
		HD	SFE
1	*α*-Pinene	0.71	0.67
2	*β*-Pinene	4.31	6.00
3	*D*-Limonene	0.2	3.55
4	*β*-Ocimene	ND	0.15
5	8-Methyl, 1-hendecene	ND	0.18
6	*α*-Guaiene	0.25	0.45
7	*β*-Caryophyllene	2.46	0.1
8	*α*-Bergamotene	ND	ND
9	3-Carene	0.46	ND
10	*α*-Cubebene	0.94	0.46
11	*α*-Muurolene	0.26	0.3
12	*γ*-Muurolene	1.67	0.2
13	*α*-Copaene	1.29	1.13
14	*β*-Bourbonene	1.97	ND
15	*β*-Guaiene	0.14	ND
	*Hydrocarbons*	**14.66**	**13.19**

16	Hinesol	0.91	2.8
17	2-Borneol	ND	0.76
18	Isopulegol	0.35	0.18
19	*trans*-Verbenol	ND	0.33
20	*cis*-Verbenol	0.46	0.95
21	Nerol	0.35	0.19
22	Geraniol	9.39	6.43
23	*trans*-Farnesol	ND	1.34
24	Geraniol	25.45	10.22
25	*trans*-Geranylgeraniol	0.4	1.31
26	3-Methyl cyclohexanol	0.71	2.01
27	*cis*-Geranylgeraniol	0.35	ND
28	Globulol	1.37	0.55
29	*γ*-Eucalyptol	ND	0.80
30	Carotol	0.37	ND
31	*α*-Cadinol	0.25	1.47
32	Cubenol	0.55	3.41
	*Alcohols*	**40.91**	**32.75**

33	Citronellal	12.77	12.57
34	Lauraldehyde	0.3	0.13
35	Neral	11.15	15.11
36	Geranialdehyde	15.12	20.02
	*Aldehydes*	**39.34**	**47.83**

37	Citronellyl acetate	ND	0.23
38	Citronellyl isobutyrate	0.61	1.27
39	Geraniol acetate	2.24	0.65
	*Esters*	**2.85**	**2.15**

40	*D*-Carvone	ND	0.15
41	*D*-Verbenone	ND	0.30
	*Ketones*	**0**	**0.45**

42	Hexadecane	0.19	ND
43	Dioctylmethane	ND	0.40
	*Alkanes*	**0.19**	**0.40**

44	Rose oxide	ND	0.10
45	Geranic acid	ND	0.14
46	Eugenol	0.17	0.33
47	2-Epoxy-*trans*-*p*-menthane	ND	0.39
48	Caryophyllene oxide	1.57	0.41
49	3-Decyne	0.19	ND
	*Others*	**1.74**	**1.37**
*Total identified*	99.69	98.14	

ND, not detected.

**Table 5:** Half-inhibition (IC_50_) values of antioxidant activities measured using DPPH radical scavenging and 1,2,3-phentriol self-oxidation.

Samples	IC_50_ (mg/g)	
	1,2,3-Phentriol self-oxidation	DPPH
SDE	274 ± 2	10 ± 1
SFE	244 ± 3	9 ± 2
AA^*∗*^	5137 ± 4	2213 ± 4
BHT^*∗*^	8029 ± 1	895 ± 3

^*∗*^Control.

**Table 6:** Minimal inhibitory concentration (MIC) of the essential oils with various extraction processes.

Immature stages	Inhibition zones (mm)				MIC (mg/mL)			
	*S. aureus*	*E. coli*	*A. niger*	*A. fumigatus*	*S. aureus*	*E. coli*	*A. niger*	*A. fumigatus*
SFE	17.8 ± 1.2	25.2 ± 0.9	14.1 ± 1.0	33.1 ± 1.3	5.6 ± 0.2	1.4 ± 0.1	2.8 ± 0.2	5.6 ± 0
SDE	14.2 ± 1.1	19.6 ± 1.2	23.1 ± 1.1	24.3 ± 1.3	5.6 ± 0.1	2.8 ± 0.2	1.4 ± 0.1	2.8 ± 0.2
Citral^*∗*^	22.2 ± 0.6	18.7 ± 1.0	19.5 ± 3.4	14.6 ± 2.3	11.2 ± 0.5	11.2 ± 0.3	5.6 ± 0.1	11.2 ± 0.1

^*∗*^Control.

## Data Availability

The data used to support the findings of this study are available from the corresponding author upon request.

## References

[1] Pavela R. (2015). Essential oils for the development of eco-friendly mosquito larvicides: a review. *Industrial Crops and Products*.

[2] Sargenti S. R., Lanças F. M. (1997). Supercritical fluid extraction of *Cymbopogon citratus* (DC.) Stapf. *Chromatographia*.

[3] Sriramavaratharajan V., Stephan J., Sudha V., Murugan R. (2016). Leaf essential oil of Cinnamomum agasthyamalayanum from the Western Ghats, India-A new source of camphor. *Industrial Crops and Products*.

[4] Pavela R., Stepanycheva E., Shchenikova A., Chermenskaya T., Petrova M. (2016). Essential oils as prospective fumigants against *Tetranychus urticae* Koch. *Industrial Crops and Products*.

[5] Calo J. R., Crandall P. G., O’Bryan C. A., Ricke S. C. (2015). Essential oils as antimicrobials in food systems-a review. *Food Control*.

[6] Patel S., Gogna P. (2015). Tapping botanicals for essential oils: progress and hurdles in cancer mitigation. *Industrial Crops and Products*.

[7] Huang H. G., Xiao Y. E., Tian L. Q. (2011). Comparative study on supercritical fluid extraction and steam distillation in extracting chemical constituents and relative contents of volatile oil from compatibility of *Ramulus cinnamomi* and *Rhizoma zingiberis*. *Journal of Experimental Traditional Medical Formulae (Chinese)*.

[8] Olmedo R. H., Asensio C. M., Grosso N. R. (2015). Thermal stability and antioxidant activity of essential oils from aromatic plants farmed in Argentina. *Industrial Crops and Products*.

[9] Arranz E., Jaime L., López de las Hazas M. C., Reglero G., Santoyo S. (2015). Supercritical fluid extraction as an alternative process to obtain essential oils with anti-inflammatory properties from marjoram and sweet basil. *Industrial Crops and Products*.

[10] Berna A., Tárrega A., Blasco M., Subirats S. (2000). Supercritical CO_2_ extraction of essential oil from orange peel; effect of the height of the bed. *Journal of Supercritical Fluids*.

[11] Baratta M. T., Dorman H. J. D., Deans S. G., Figueiredo A. C., Barroso J. G., Ruberto G. (2015). Antimicrobial and antioxidant properties of some commercial essential oils. *Flavour and Fragrance Journal*.

[12] Dorman H. J. D., Figueiredo A. C., Barroso J. G., Deans S. G. (2015). In vitro evaluation of antioxidant activity of essential oils and their components. *Flavour and Fragrance Journal*.

[13] Niu C. Y., Zhang F. Q., Diao D. M., Yan F. U. (2014). Extraction conditions optimization of essential oil from steamed ginseng water by response surface methodology and GC-MS analysis. *Science and Technology of Food Industry (Chinese)*.

[14] Harkat M. L., Asma B., Madani K. (2015). Chemical composition, antibacterial and antioxidant activities of essential oil of *Eucalyptus globulus* from Algeria. *Industrial Crops and Products*.

[15] Kil H. Y., Seong E. S., Ghimire B. K. (2009). Antioxidant and antimicrobial activities of crude sorghum extract. *Food Chemistry*.

[16] Rakmai J., Cheirsilp B., Torrado-Agrasar A., Simal-Gándara J., Mejuto J. C. (2017). Encapsulation of yarrow essential oil in hydroxypropyl-beta-cyclodextrin: physiochemical characterization and evaluation of bio-efficacies. *CyTA-Journal of Food*.

[17] Rakmai J., Cheirsilp B., Mejuto J. C., Simal-Gándara J., Torrado-Agrasar A. (2018). Antioxidant and antimicrobial properties of encapsulated guava leaf oil in hydroxypropyl-beta-cyclodextrin. *Industrial Crops and Products*.

[18] Rakmai J., Cheirsilp B., Mejuto J. C., Torrado-Agrasar A., Simal-Gándara J. (2016). Physico-chemical characterization and evaluation of bio-efficacies of black pepper essential oil encapsulated in hydroxypropyl-beta-cyclodextrin. *Food Hydrocolloids*.

[19] Carlson L. H. C., Machado R. A. F., Spricigo C. B., Pereira L. K., Bolzan A. (2001). Extraction of lemongrass essential oil with dense carbon dioxide. *Journal of Supercritical Fluids*.

[20] Qiu Q., LIing J., Ding Y., Chang H., Wang J., Liu T. (2005). Comparison of supercritical fluid extraction and steam distillation methods for the extraction of essential oils from *Schizonepeta tenuifolia* briq. *Chromatography*.

[21] Sodeifian G., Sajadian S. A., Saadati Ardestani N. (2017). Experimental optimization and mathematical modeling of the supercritical fluid extraction of essential oil from *Eryngium billardieri* : application of simulated annealing (SA) algorithm. *Journal of Supercritical Fluids*.

[22] Gasparetto A., Bella Cruz A., Wagner T. M., Bonomini T. J., Correa R., Malheiros A. (2017). Seasonal variation in the chemical composition, antimicrobial and mutagenic potential of essential oils from Piper cernuum. *Industrial Crops and Products*.

[23] Konoz E., Hajikhani N., Abbasi A. (2017). Comparison of two methods for extraction of dill essential oil by gas chromatography-mass spectrometry coupled with chemometric resolution techniques. *International Journal of Food Properties*.

[24] Zhang H. J., Cao Y. B., Xue W. B., Ding H., Liu G. F., Wang J. Q. (2017). Optimization of extraction process of citronella essential oil and its gas phase antibacterial properties. *China Condiment*.

[25] Guillen M. D., Cabo N., Burillo J. (1996). Characterisation of the essential oils of some cultivated aromatic plants of industrial interest. *Journal of the Science of Food and Agriculture*.

[26] Hu J., Nie S., Huang D., Li C., Xie M. (2012). Extraction of saponin from *camellia oleifera* cake and evaluation of its antioxidant activity. *International Journal of Food Science and Technology*.

[27] Singh J., Tripathi N. N. (2015). Inhibition of storage fungi of blackgram (*Vigna mungo* L.) by some essential oils. *Flavour and Fragrance Journal*.

[28] Matusinsky P., Zouhar M., Pavela R., Novy P. (2015). Antifungal effect of five essential oils against important pathogenic fungi of cereals. *Industrial Crops and Products*.

